# Host surface ectonucleotidase-CD73 and the opportunistic pathogen, *Porphyromonas gingivalis*, cross-modulation underlies a new homeostatic mechanism for chronic bacterial survival in human epithelial cells

**DOI:** 10.1080/21505594.2020.1763061

**Published:** 2020-05-18

**Authors:** Jaden S. Lee, Nityananda Chowdhury, JoAnn S. Roberts, Özlem Yilmaz

**Affiliations:** a Department of Oral Health Sciences, Medical University of South Carolina, Charleston, SC, USA; b Department of Microbiology and Immunology, Medical University of South Carolina, Charleston, SC, USA

**Keywords:** Ectonucleotidase-CD73, purinergic signaling, opportunistic oral bacteria, epithelial cells, intracellular infection, persistence

## Abstract

Cell surface nucleotide-metabolizing enzyme, ectonucleotidase-CD73, has emerged as a central component of the cellular homeostatic-machinery that counterbalances the danger-molecule (extracellular-ATP)-driven proinflammatory response in immune cells. While the importance of CD73 in microbial host fitness and symbiosis is gradually being unraveled, there remains a significant gap in knowledge of CD73 and its putative role in epithelial cells. Here, we depict a novel host-pathogen adaptation mechanism where CD73 takes a center role in the intracellular persistence of *Porphyromonas gingivalis*, a major colonizer of oral mucosa, using human primary gingival epithelial cell (GEC) system. Temporal analyses revealed, upon invasion into the GECs, *P. gingivalis* can significantly elevate the host-surface CD73 activity and expression. The enhanced and active CD73 significantly increases *P. gingivalis* intracellular growth in the presence of substrate-AMP and simultaneously acts as a negative regulator of reactive oxygen species (ROS) generation upon eATP treatment. The inhibition of CD73 by siRNA or by a specific inhibitor markedly increases ROS production. Moreover, CD73 and *P. gingivalis* cross-signaling significantly modulates pro-inflammatory interleukin-6 (IL-6) in the GECs. Conversely, exogenous treatment of the infected GECs with IL-6 suppresses the intracellular bacteria via amplified ROS generation. However, the decreased bacterial levels can be restored by overexpressing functionally active CD73. Together, these findings illuminate how the local extracellular-purine-metabolism, in which CD73 serves as a core molecular switch, can alter intracellular microbial colonization resistance. Further, host-adaptive pathogens such as *P. gingivalis* can target host ectonucleotidases to disarm specific innate defenses for successful intracellular persistence in mucosal epithelia.

## Introduction

Epithelial cells are the first-line innate defense cells that invading microbes encounter, therefore how this interaction develops can determine the colonization potential of a microorganism and its severity and duration in the host[1, 2]. In the oral cavity, gingival epithelial cells are centrally essential for initial proper induction of mucosal immune response since these cells often can recognize and respond to pathogens without causing serious damage to the host [1,3–5]. The mucosal epithelia actively participate in the immune defense via transfer of intracellular molecules to the extracellular space, which signals probable danger to the immune cells [6,7]. With starting infection or cellular stress, the host cells release adenosine 5ʹ-triphosphate (ATP) through pannexin-1-hemichannel to the extracellular milieu, thereby modulating and possibly disrupting the homeostatic purinergic signaling [8,9]. The host-derived small danger molecules then can act on ionotropic purinergic receptors, particularly P2X_7_ receptor, to elicit specific pro-inflammatory signal transduction [10–13]. To counteract the increase in the pro-inflammatory effector extracellular ATP (eATP), a series of host ectonucleotidases dynamically regulate rapid phosphohydrolysis of these nucleotides. Most notably, the production of extracellular adenosine occurs primarily through CD73 (ecto-5ʹ-nucleotidase), a glycosylphosphatidylinositol (GPI)-linked, membrane-bound enzyme that hydrolyzes extracellular nucleoside di/monophosphates into bioactive intermediates [14]. CD73-generated adenosine subsequently can lead to activation of G-protein-coupled adenosine receptors, which later regulate diverse physiologic effects including anti-inflammatory actions [15]. CD73 is known as a ubiquitous enzyme [16]. Expression of CD73 may change dynamically in cancer and chronic inflammatory conditions mostly examined in professional immune cells for its potent immunosuppressive functions [17–23]. However, the presence of CD73 has not been well characterized in epithelial cells and never been studied in the oral epithelia. Only recently is CD73 noted as a putative modulator in regulation of specific immune responses against microbial colonization [9,24] whilst CD73-mediated generation of extracellular adenosine signaling in epithelial cells remains largely unexplored [9].

The oral mucosa is a dynamic ecosystem with more than 700 species of intricately connected microorganisms [25,26]. Among those, *Porphyromonas gingivalis*, a Gram-negative bacterium and facultative intracellular pathogen, has been named a keystone organism for its major contribution to progression of periodontal disease and dysbiotic microbiota [27,28]. Recently, *P. gingivalis* has been proposed as an etiologic factor in various other chronic diseases, including orodigestive cancers and Alzheimer’s disease [29–31]. In gingival epithelial cells (GECs), *P. gingivalis* can establish its intracellular replication niche/reservoir [32–34] and later spread to adjacent cells intercellularly as a means of evading host antimicrobial immune detection [35] during disseminating deeper within the tissue [3,35–39]. Upon invasion into GECs, *P. gingivalis* can facilitate a long-term survival by altering host danger signal eATP-induced pathways that result in specific intracellular events such as modulation of reactive oxygen species (ROS) generation and pro-inflammatory cytokine Interleukin-1β (IL-1β) secretion [3,37,39–42]. Further, *P. gingivalis* inhibits GEC cell death induced by various pro-inflammatory or pro-apoptotic molecules [1,32,37,39,43,44]. By remaining viable in these host cells without being cleared, *P. gingivalis* forms a chronic infection in the oral mucosa, which can subsequently drive microorganismal proliferation/survival as well as dysbiosis in the oral microbiota [45]. Despite the past and ongoing endeavors, it is unclear under what microenvironmental deviations and molecular signals *P. gingivalis* gains supremacy over innate cellular defenses for a successful chronic microbial establishment in the oral mucosa. The significance of the purinergic signaling, which involves danger signals eATP and adenosine, has lately grown strong for colonization of opportunistic pathogens such as *P. gingivalis* in the epithelial mucosa [46–48]. Increasing evidence also supports the role of adenosine for progression of chronic inflammatory diseases [49]. Recent reports have investigated involvement of adenosine signaling in periodontal disease [50–52]. A study using rat models showed adenosine-dependent reduction in oral inflammation [52,53]. Moreover, we have previously shown that the purine signaling is critical for *P. gingivalis* in modulation of IL-1β [41] and that primary GECs express all types of adenosine (A_a_) receptors including A2a with anti-inflammatory downstream effects including cAMP generation [54]. Addition of A2a receptor-specific agonist to *P. gingivalis*-infected GECs resulted in more intracellular *P. gingivalis*, whereas decreased bacterial growth was found with A2a receptor-specific antagonist, suggesting the pathogen may usurp the danger signal adenosine-coupled A2a signaling for intracellular life [54].

In the present work, using human primary GECs as a biologically relevant reductionist model [55–57], we report critically novel facets of CD73 in modulating epithelial-cell antimicrobial defenses via extracellular-purine-nucleotides. Specifically, we demonstrate for the first time the ectonucleotidase-CD73 expression and activity in the human primary epithelial cells, both of which are significantly increased during the opportunistic pathogen, *P. gingivalis* infection. We further show that the enhanced CD73 activity also coupled by extracellular AMP availability during the infection can be vital for the intracellular bacterial growth in epithelial cells. Interestingly, CD73 can play a crucial role for cross-modulation of select epithelial innate responses by *P. gingivalis*, including the danger signal eATP-generated reactive oxygen species (ROS) and dysregulated interleukin-6 (IL-6, one of the leading inflammatory mediators in a chronic inflammatory human disease, periodontitis). Lastly, we reveal that the intracellular level of *P. gingivalis* can be significantly reduced by exogenous treatment of IL-6, which can be largely restored by overexpressing CD73 in GECs. These findings together allude a novel host-pathogen adaptation mechanism specifically mediated by the host homeostatic CD73 and *P. gingivalis* interaction in oral mucosal cells. The targeting of CD73 by *P. gingivalis* can aid the microorganism forming a strategic growth-favorable cellular niche with the weakened actions of innate antibacterial molecules (e.g. ROS and IL-6). The described complex interaction may have a direct bearing on the dysbiotic presence of this keystone pathogen in human mucosa and could be an important mechanism used by other successful persistent pathogens.

## Results

### Examining the expression of ectonucleotidase-CD73 in GECs and its induction by P. gingivalis infection

**Figure 1.**
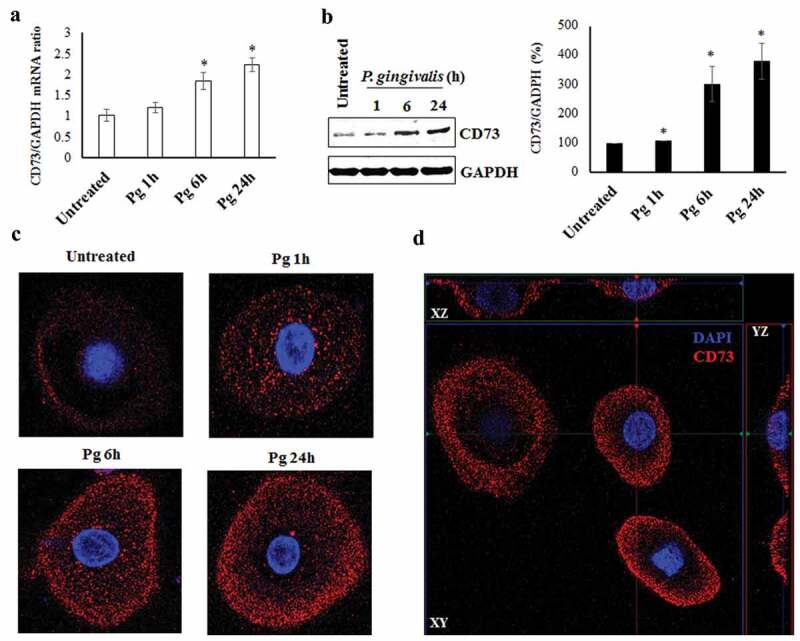
Ectonucleotidase-CD73 expression in primary GECs significantly increases during *P. gingivalis* infection. a) *P. gingivalis* strain ATCC 33277 was added at MOI 100 to cultured GECs over 24 h. GAPDH was used as a normalization control. SybrGreen detection of mRNA expression levels of CD73 (nt5e) using qRT-PCR. N = 3, *p < 0.05 as compared to untreated; Mean ± SEM. b) Infection was performed in the same manner as a). Cell lysates were extracted and immunoblotted with a CD73 antibody. Densitometric analysis was performed using NIH ImageJ software. N = 3, *p < 0.05 as compared to untreated. Mean ± SEM. c) Cells with *P. gingivalis* infection over 24 h were fixed, stained with primary antibody against CD73 and then secondary antibody (Alexa fluor 468; red), and mounted with DAPI (blue) to visualize nuclei. Representative images were obtained via confocal microscopy at 63x objective with oil immersion. d) Confocal micrographs showing orthogonal views of surface CD73 expression in GECs.

We initially examined via qRT-PCR and Western blotting the expression of ectonucleotidase CD73 in *P. gingivalis* infected GECs over 24 h post-infection and compared the levels with uninfected GECs. Our results showed that both mRNA (Figure 1(a)) and protein (Figure 1(b)) expression of CD73 was significantly increased at 6 h post-bacterial invasion and remained gradually increased over 24 h of *P. gingivalis* infection. Further examination using confocal microscopy with specifically immuno-stained GECs also depicted significantly increased CD73 expression during infection (Figure 1(c), S1A). We demonstrated the exclusive external localization of CD73 on the cell membranes through immuno-staining (red) by orthogonal views of GECs (Figure 1(d), S1B). These data together indicate that *P. gingivalis* induces gradual and significantly higher CD73 expression in GECs during infection.

### Examining the biological activity of ectonucleotidase-CD73 in GECs with or without P. gingivalis infection

To further validate the functional presence of CD73 in human gingival epithelial cells, we first assessed the potency of α,β-methylene adenosine-5′-diphosphate (APCP; a CD73-specific inhibitor) [59] as well as the effect of adenosine monophosphate (AMP; specific substrate for CD73) [60] treatment on CD73 activity in human primary GECs. Cells were incubated in phosphate-free buffer in the presence or absence of APCP and AMP and the amount of free phosphate released was quantified via the malachite green assay method.

**Figure 2.**
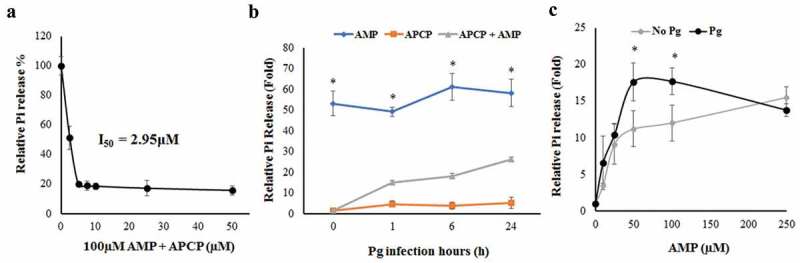
Confirmation of functional presence of the CD73 and the effect of *P. gingivalis* infection on CD73 activity in GECs. GECs were incubated in phosphate-free buffer and treated with either AMP, APCP, and/or *P. gingivalis*. All graphs (a-c) were obtained using Malachite Green Phosphate assay kit following the manufacturer’s instructions (Bioassay Systems). a) Inhibition–concentration curve showing CD73 activity of cells pre-treated with AMP [100 μM] and further incubated with varying concentrations of APCP. b) CD73 activity in untreated and *P. gingivalis* infected GECs (1, 6, and 24 h) in the presence of AMP [100 µM] and/or APCP [10 µM] N = 6, *p < 0.05 as compared for AMP vs. AMP + APCP. Mean ± SEM. c) CD73 activity with/without *P. gingivalis* infection at varying AMP concentrations. Cells were infected with *P. gingivalis* for 6 h and stimulated with AMP for 30 min post infection. N = 6, *p < 0.05 as compared to AMP 0 μM; Mean ± SEM.

The inhibitory effect of APCP on human CD73 in GECs was first determined. The concentration–inhibition curve exhibited an I_50_ value of 2.95 μM for the inhibitor in GECs pre-treated with AMP to initiate the enzymatic reaction (Figure 2(a)), showing comparable potency of the inhibitor with published data [61,62]. This suggests that ectonucleotidase-CD73 is fully functional in human primary GECs.

By the malachite green assay method, we next examined CD73 enzymatic activity in the presence of *P. gingivalis* infection using APCP and AMP treatment (Figure 2(b,c)). When GECs were infected with *P. gingivalis* to mimic the inflammatory environment, the AMP treatment alone rapidly exhibited significantly increased phosphate release as expected at ~53-fold, which confirms that AMP is a CD73-specific substrate in GECs and stimulates high conversion to adenosine resulting in increased phosphate release (Figure 2(b)). Cells treated with both APCP and AMP showed significant decrease in phosphate release compared to those treated with AMP only, confirming that APCP functions as a robust CD73 inhibitor in GECs during the infection. While uninfected GECs showed an approximately 10-fold increase in CD73 activity with 50 and 100 μM of AMP pre-treatment, this increase was further augmented by *P. gingivalis* infection showing enhanced CD73 enzymatic activity by ~17-fold when pre-treated with the same concentrations of AMP. These data collectively suggest that the epithelial cells possess functional CD73 and its activity levels can be significantly enhanced by the infection.

### Increased activity of ectonucleotidase-CD73 enhances intracellular growth of P. gingivalis in GECs

**Figure 3.**
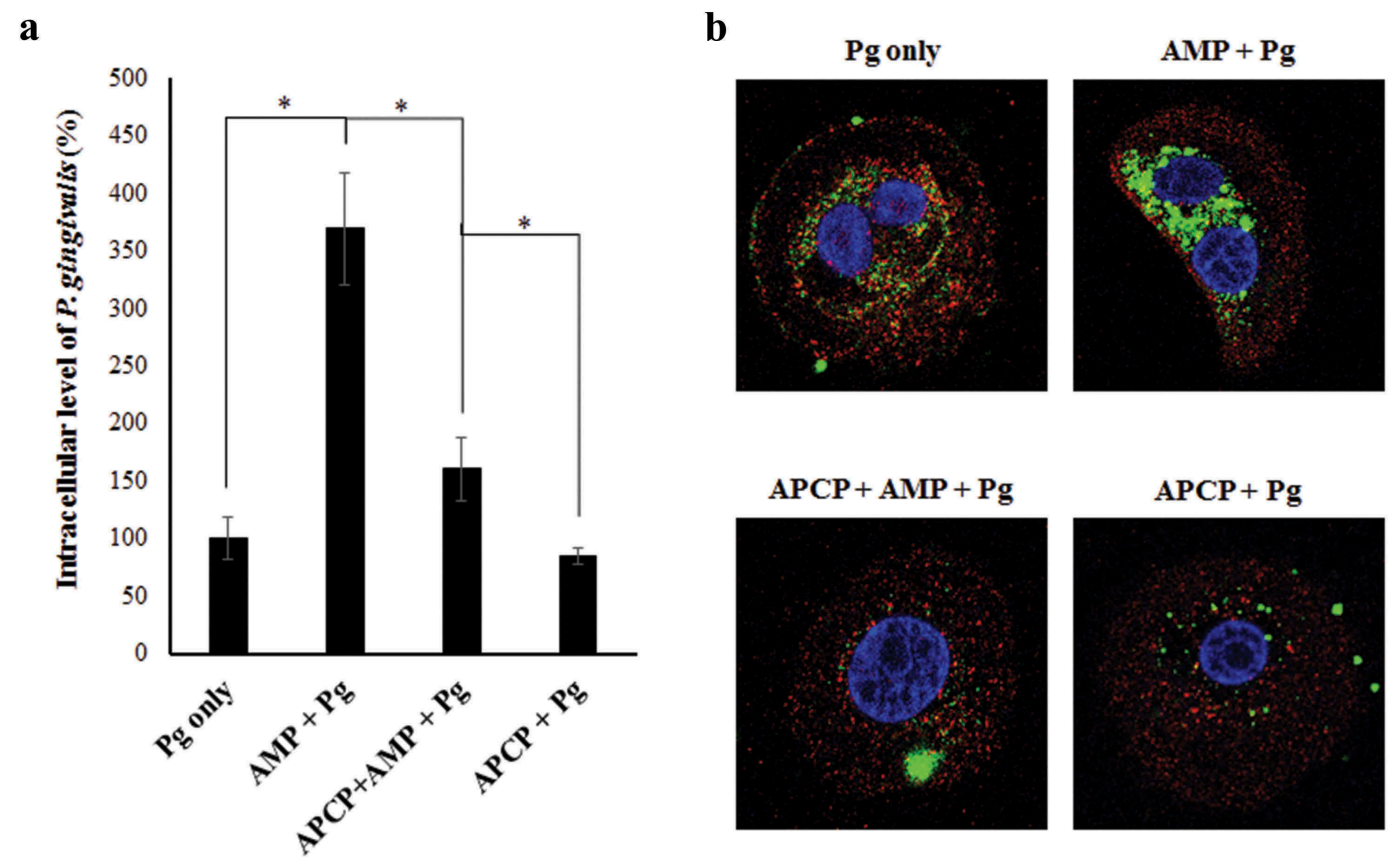
The active CD73 significantly increases intracellular *P. gingivalis* levels in GECs. a) Antibiotic protection assay – GECs were infected with *P. gingivalis* (MOI 100) for 24 h. APCP [50 μM] and AMP [100 μM] were added to cells 1 h and 30 min prior to infection, respectively. Cells were then incubated with metronidazole [200 μg/mL] and gentamicin [300 μg/mL] for an additional 1 h. RNAs from each sample were isolated using Trizol and subsequently obtained cDNAs were subjected to qPCR analysis using *P. gingivalis* 16s rRNA primers. N = 3, *p < 0.05; Mean ± SEM. b) Representative confocal microscopy images of fixed, infected GECs with *P. gingivalis* (MOI 100) for 24 h with or without AMP and APCP as described in a). CD73 (red) and *P. gingivalis* (green) were visualized by immunostaining. 63x magnification with oil immersion. The range of z-stacks was kept consistent.

We next evaluated whether CD73 can play a specific role in *P. gingivalis* survival in GECs. Accordingly, we examined the intracellular survival of *P. gingivalis* in GECs in the presence of CD73 substrate molecule AMP or the CD73 inhibitor APCP using our *in-situ* antibiotic protection assay [63]. Cells were first incubated with or without 50 μM APCP followed by 100 μM AMP stimulation and 24 h infection with *P. gingivalis*. We then quantified live, intracellular bacteria by qPCR using 16s rRNA primers (Figure 3(a)). The results showed more than a 3.5-fold increase in the intracellular P. *gingivalis* levels in GECs treated with AMP compared to the AMP untreated cells only infected with *P. gingivalis*. Infected cells treated with APCP in addition to AMP stimulation showed significantly reduced bacterial survival than the infected cells treated with AMP only. Interestingly, APCP treatment alone exhibited a significant decrease only when compared to the infected cells treated with both APCP and AMP (Figure 3(a)). Compared to the control cells with only infection, APCP treatment did not have a notable impact on bacterial levels, which points to the importance of AMP as an initiator of the enzymatic reaction. To further confirm our findings via visualization, we performed immunostaining on *P. gingivalis* infected GECs and analyzed the levels of intracellular bacteria through confocal microscopy (Figure 3(b)). *P. gingivalis* specifically immuno-stained in green fluorescence is significantly increased in cells treated with AMP compared to the control cells with infection only as well as the host cells further treated with APCP (Figure 3(b)). These findings suggest that high CD73 activity, which was experimentally mimicked by exogenous addition of AMP to cell culture milieu, can be essential for *P. gingivalis* to establish an optimal level of intracellular growth and survival in the epithelial cells of oral mucosa.

### Ectonucleotidase-CD73 is a negative regulator of a danger signal eATP induced ROS production in GECs

**Figure 4.**
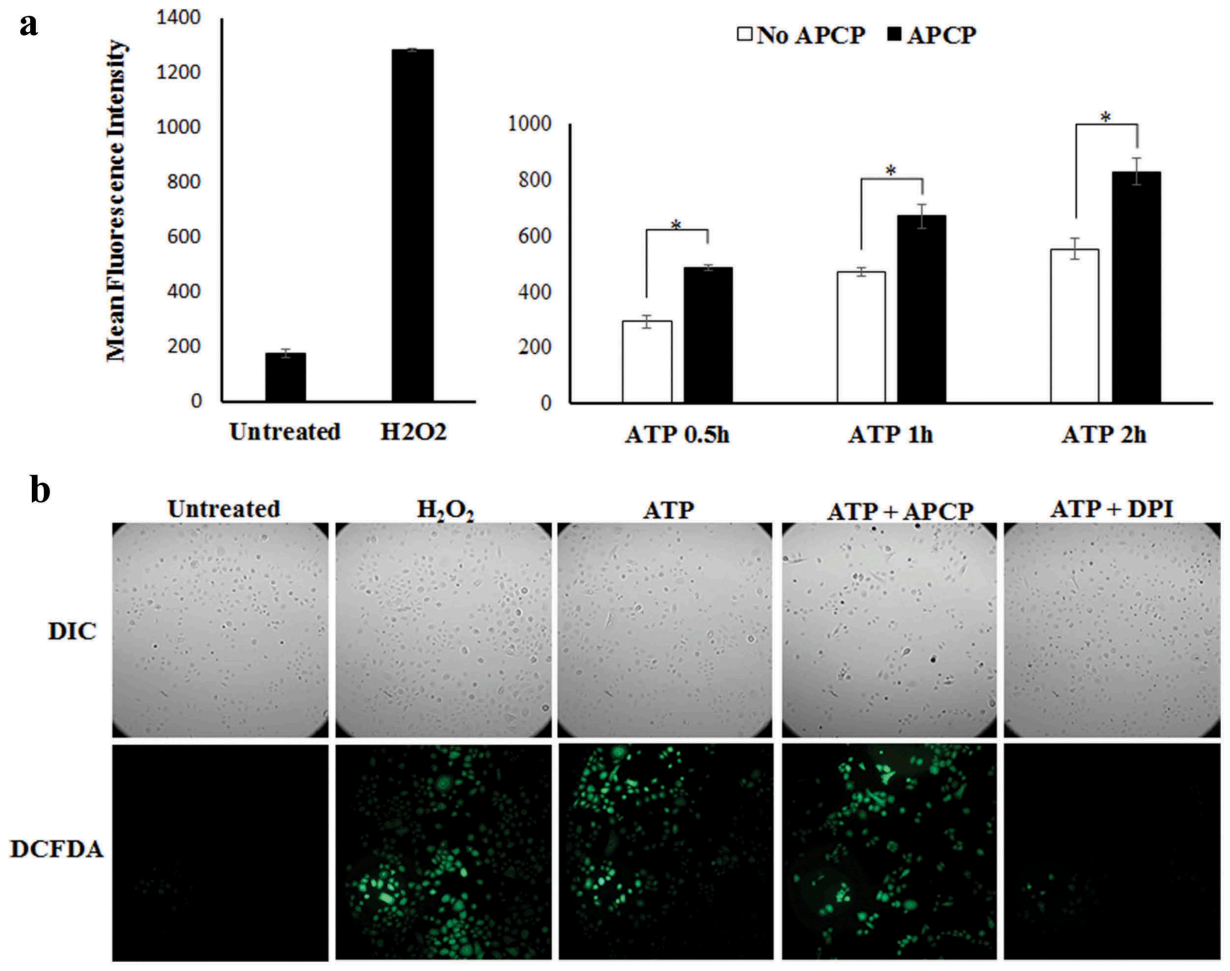
The CD73 significantly diminishes eATP induced reactive oxygen species (ROS) generation in GECs. a) ROS generation in GECs after 30 min pre-treatment with ATP [3 mM] and incubation over 2 h with APCP [50 μM] or vehicle measured as relative fluorescence using a Biotek H1 M monochromatic plate reader at 525 nm. N = 3, *p < 0.05; Mean ± SEM. b) Representative micrographs of CM-H_2_DCFDA, ROS probe, live fluorescence imaging with Differential Interference Contrast (DIC) microscopy was used for qualitative assessment of ROS generation shown in a). 10x magnification on Leica inverted microscope. Diphenyleneiodonium (DPI), an NAD(p)H oxidase inhibitor, was used as a positive control at 50 μM.

Our previous studies showed that *P. gingivalis* can attenuate eATP-induced biocidal ROS production [3,39] and CD73 may regulate ROS levels in cells or tissues [64,65]. Therefore, we assessed whether CD73 specifically involves in modulation of eATP coupled ROS production in GECs using a ROS-detecting fluorescent probe, DCFDA, for *in-situ* quantitative and qualitative ROS analysis. Diphenyleneiodonium (DPI; a broad-spectrum NADPH oxidase inhibitor) and hydrogen peroxide (exogenous ROS) were used as controls and underwent the probe incubation in the same manner. Treatment of eATP-stimulated GECs with the CD73 inhibitor APCP significantly increased eATP-induced ROS as early as 30 min after initial ATP treatment (Figure 4(a)). This large increase in ROS levels in the presence of APCP was vigorous and sustained, suggesting the involvement of CD73 in decreasing of cellular ROS produced by the GECs in response to eATP. Live cell imaging for cellular ROS showed that inhibiting CD73 in GECs by APCP also resulted in notably greater ROS production than the cells stimulated with eATP only (Figure 4(b)). Taken together, these results suggest that CD73 perhaps is an important negative regulator of danger signal eATP-induced antimicrobial ROS in GECs.

### Activation of ectonucleotidase-CD73 by P. gingivalis decreases host cell-mediated induction of antimicrobial interleukin-6 (IL-6) in GECs

**Figure 5.**
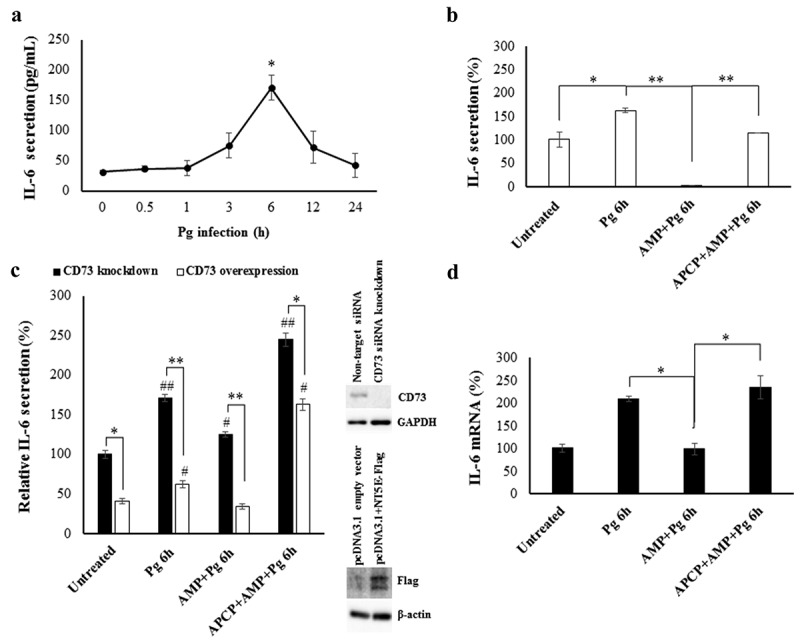
The active and enhanced CD73 attenuates human IL-6 expression and secretion induced by GECs during *P. gingivalis* infection. a) Quantitative ELISA measurement of human IL-6 expression in GECs with *P. gingivalis* infection (MOI 100) over 24 h displayed in μM. N = 3, *p < 0.05 as compared to untreated; Mean ± SEM. b,c) Quantitative ELISA measurement of human IL-6 secretion in untreated GECs, CD73 knockdown, and overexpressing GECs incubated with *P. gingivalis* (MOI 100) for 6 h. Further treatment with APCP [50 μM] and/or AMP [100 μM] was also performed. CD73 depletion/overexpression studies were conducted using non-target siRNA control and empty vector as controls and confirmed by Western blotting. N = 3, #,*p < 0.05, ##,**p < 0.001, # denotes statistical comparison to corresponding untreated; Mean ± SEM. d) GECs were pre-infected with *P. gingivalis* (MOI 100) for 6 h, treated with APCP [50 μM] and/or AMP [100 μM], and analyzed by qPCR using SYBR Green for IL-6 mRNA levels. N = 3, *p < 0.05; Mean ± SEM.

Recent studies have shown modulation of IL-6 by ROS accumulation [66,67]. Hence, we next investigated the levels of IL-6, during *P. gingivalis* infection and whether CD73 plays a role in regulating the expression and/or secretion of IL-6 in GECs. We first measured the effect of *P. gingivalis* infection on levels of IL-6 secretion over 24 h (Figure 5(a), S2). The infection by *P. gingivalis* markedly modulated IL-6 secretion with a steady and significant increase at 6 h post-infection, which also resulted in a large decline returning into baseline IL-6 levels at 24 h post-infection. In addition to our published studies which had already demonstrated that *P. gingivalis* infection does not induce neither apoptosis nor necrosis in primary GECs and the cells are viable at least up to 120 h post-infection [1,3,35,37,40,63,68], we further confirmed that the marked decrease in IL-6 production after 6 h post-infection is not due to GEC cell death (Figure S3).

Subsequently, we examined whether CD73 has any regulatory effect on IL-6 secretion during *P. gingivalis* infection. ELISA analysis showed that the initial IL-6 secretion produced in GECs by *P. gingivalis* is significantly weakened upon CD73 stimulation by exogenous AMP. Further, inhibition of CD73 activity by APCP significantly diminished the suppressive effect of AMP (Figure 5(b)). As additional investigations, CD73-specific gene depletion via siRNA or overexpression in GECs was performed (Figure 5(c)). CD73-depleted GECs showed significantly increased IL-6 secretion for corresponding conditions tested compared to CD73-overexpressing cells, indicating that levels of CD73 are also important for attenuating the pro-inflammatory IL-6 production (Figure 5(c)). These results collectively highlight a dynamically specific molecular dialogue between the host CD73 and the microorganism that culminates in reduction of IL-6 production. Interestingly, qRT-PCR analysis for IL-6 gene expression at 6 h of *P. gingivalis* infection in GECs displayed IL-6 mRNA expression is significantly downregulated by CD73 stimulation with AMP treatment, which is fully rescued by CD73 inhibition with APCP (Figure 5(d)), a similar trend observed in the protein level (Figure 5(b)). This finding further points to a tight regulation of IL-6 by CD73 pathway that may also occur at the IL-6 gene level.

### Ectonucleotidase-CD73-mediated inhibition of eATP-coupled ROS generation is critical for IL-6 production in GECs

**Figure 6.**
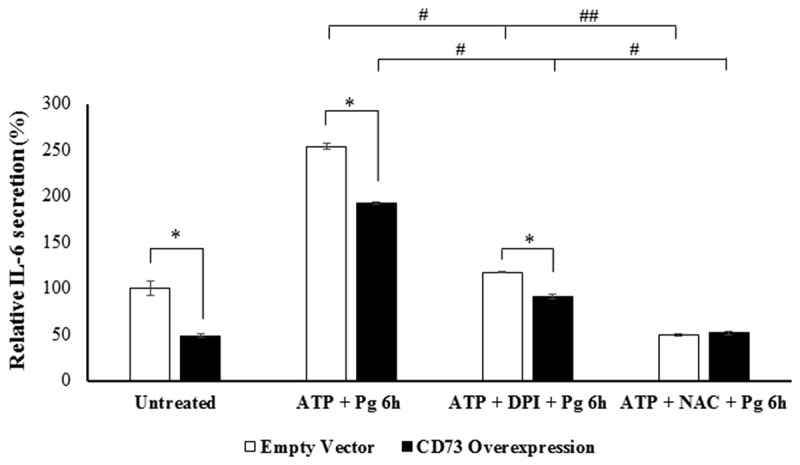
ATP-mediated ROS generation regulates IL-6 secretion in a CD73-dependent manner during *P. gingivalis* infection. Quantitative ELISA measurement of human IL-6 secretion in control GECs treated with the empty vector, and overexpressing GECs incubated with *P. gingivalis* (MOI 100) for 6 h. Further treatment with ATP [3 mM], a broad-spectrum NADPH oxidase inhibitor DPI [50 μM] and/or ROS scavenger NAC [50 μM] was also performed. N = 6, *p < 0.05, #p < 0.05, ##p < 0.001; Mean ± SEM.

We next examined the plausible role of CD73-mediated ROS for IL-6 secretion in GECs during *P. gingivalis* infection. Besides the untreated cells, all GECs were pre-stimulated with eATP for ROS induction and infected with *P. gingivalis* for 6 h. Some cells were further incubated with either DPI or N-acetyl-l-cysteine (NAC; potent antioxidant) to suppress ROS formation and accumulation, respectively. For both control and CD73-overexpressing GECs, eATP treatment resulted in significantly higher IL-6 secretion levels compared to those when further treated with the ROS inhibitors (Figure 6). More critically, overexpression of CD73 in GECs led to significantly reduced amount of secreted IL-6 when cells were either treated with ATP only or ATP and DPI together. Interestingly, NAC treatment seemed to induce a more robust inhibition of IL-6 secretion than DPI, which suggests the putative link between intracellular ROS and IL-6 secretion may not be entirely limited to membrane-associated NADPH oxidase derived ROS. Overall, these findings support that CD73 can be a key determinant for IL-6 secretion stimulated by the danger signal eATP-mediated ROS production in GECs upon infection.

### Exogenous IL-6 treatment reduces the intracellular level of P. gingivalis and the Ectonucleotidase-CD73 overexpression can restore the IL-6-mediated decrease in P. gingivalis survival

**Figure 7.**
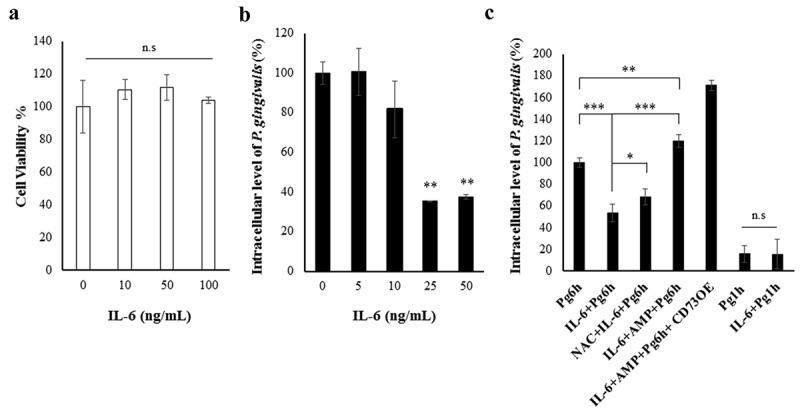
CD73 overexpression reverses the decreasing *P. gingivalis* intracellular level in GECs caused by exogenous treatment of human IL-6. a) Cells were incubated with multiple doses of IL-6 for 24 h. GEC cell viability was assessed using MTT assay. n.s = no statistically significant difference, N = 3, * p < 0.05. For B and C, GECs were infected with *P. gingivalis* (MOI 100) for 6 h. b) Human recombinant active IL-6 [25 ng/mL] was added to cells 30 min prior to infection at varying concentrations. IL-6 treatment [25 ng/mL] was performed similarly in B and C. c) Overexpression of CD73 (OE) was implemented in some conditions as described in *Material & Methods*. For B and C, cells were later incubated with metronidazole [200 μg/mL] and gentamicin [300 μg/mL] for an additional 1 h to kill extracellular bacteria. RNAs from each sample were isolated using Trizol and subsequently obtained cDNAs were subjected to qPCR analysis using *P. gingivalis* 16s rRNA primers. N = 6, *p < 0.05, **p < 0.001, *** <0.0001; Mean ± SEM. CD73OE = CD73 overexpression, NAC = N-acetyl-l-cysteine (ROS scavenger; 50 µM).

Given the putative role of CD73 for promoting intracellular *P. gingivalis* growth and inhibiting eATP-mediated antimicrobial IL-6 secretion during infection in GECs, we studied the potential direct effect of IL-6 on intracellular *P. gingivalis* survival. Accordingly, we tried to emulate the pro-inflammatory environment by treating the host cells exogenously with human IL-6 in addition to the CD73-specific substrate (AMP). We first established that extracellular addition of human IL-6 does not induce host cell toxicity in GECs via time-course MTT assay and the bacterial decrease would not be the result of host cell death (Figure 7(a)). We next examined the effect of exogenous IL-6 treatment at varying concentrations on intracellular *P. gingivalis* levels in GECs at 6 h post-infection where we observed the highest IL-6 production during the infection (Figure 7(b)). Our results showed that exogenous IL-6 treatment substantially decreased the bacterial survival in a dose-dependent manner, exhibiting a significant decrease at 25 and 50 ng/mL.

Further, we re-performed the antibiotic protection assay, using *P. gingivalis-*infected GECs pre-treated with IL-6 as previously done in Figure 7(b). However, we this time amplified the activity of CD73 in two ways: AMP treatment alone and AMP treatment plus CD73 overexpression (Figure 7(c)). Results showed that AMP treatment alone not only significantly restored the IL-6 induced reduction in the intracellular *P. gingivalis* survival but also presented unexpected synergy by inducing significantly more bacteria compared to the baseline *P. gingivalis* 6 h levels in GECs. Additional CD73 overexpression accompanied by AMP treatment significantly rescued and further enhanced the bacterial replication, yielding highest (more than a 70%) increase compared to the GECs incubated with *P. gingivalis* only. Interestingly, additional treatment with a potent ROS scavenger NAC showed a substantial reversion on the antibacterial property of IL-6 compared to that GECs treated with IL-6 only (﻿the NADPH oxidase inhibitor DPI showed a similar reversing effect; data not shown). This result further supports that IL-6 may exert an inhibitory effect on intracellular *P. gingivalis* survival through ROS induction. We also confirmed no interference of initial bacterial invasion by IL-6 treatment (Figure 7(c), invasion percentage with and without IL-6 treatments at 1 h post-infection is comparable). These findings together describe a new molecular circuitry where the opportunistic *P. gingivalis* can reprogram the host anti-inflammatory CD73 signaling to disarm the antibacterial effect of high IL-6 levels initially induced by the GECs as a response to growing infection.

## Discussion

Increasing studies have highlighted the importance of host-derived small molecules, such as ATP and adenosine, which function as a danger signal in host and alert the immune system to properly respond to threat via purinergic signaling [69–71]. Extracellular ATP accumulation has been strongly associated with upregulation of pro-inflammatory immune responses including cytokine and cellular ROS levels [72]. On the contrary, adenosine is a well-established inflammatory mediator with immunosuppressive actions including inhibition of tumor growth and regulation of Treg cells [73,74]. As a major generator of adenosine, CD73 in various specialized immune cells performs several innate protective functions important for various severe inflammatory diseases [19,21,74]. Growing evidence suggests that CD73 may modulate opportunistic bacterial colonization in host tissues [9,18,21,75]. CD73 also appears to be an important determinant of the extracellular homeostasis for maintaining the physiological state of host cells in the face of potent danger signaling molecules. This distinct ability of CD73 may present a greater degree of importance to host-adapted chronic pathogens such as *P. gingivalis* for promoting cellular growth and survival, which may later create dysbiotic microbiota. Several lines of *in vivo* studies have shown that CD73 can impact on various types of microbial infection in the intestinal mucosa through the modulation of adaptive immune responses [18,76,77]. *Salmonella-*treated murine splenocytes incubated with a CD73-specific inhibitor APCP showed significantly augmented IL17A and IFN-γ expression [21]. Liver tissue from CD73-deficient mice also had attenuated bacterial load of *Salmonella*, which collectively suggests CD73 expression can exacerbate the outcome of *Salmonella* infection by regulating host inflammatory responses [21]. Furthermore, these recent studies merely utilized CD73 knockout mice to phenotypically describe and establish the initial importance of this enzyme for mucosal immunity during acute and chronic models of microbial infection [18,21,76,78,79].

Here, we present a newly identified expression of functional CD73 in the human primary epithelial cells and CD73’s novel well-orchestrated molecular actions in regulating a cellular chronic infection by the opportunistic pathogen, *P. gingivalis*. Both mRNA and protein expression of CD73 is markedly increased by *P. gingivalis* infection, indicating that the infection acts as a robust danger signal for initiation of the CD73 enzymatic reaction. Furthermore, the immunostaining of CD73 visually confirmed that *P. gingivalis* infection increases the expression of surface-bound CD73 in GECs. Our results from these expression studies provide a first direct visualization of the interaction between an opportunistic bacterium and CD73 in host epithelial cells.

To date, the primary known action of CD73 is to metabolize AMP to produce adenosine by hydrolyzing nucleotide monophosphates while releasing inorganic phosphate, which have been shown in various immune cells [14,76]. However, no prior studies have confirmed this function in human epithelial cells. While making sure that the concentration of AMP used in the experiments was within the biologically analogous range (i.e. 50–100 μM; AMP higher than 250 μM is considered physiologically high) [80], our novel results showed further increased CD73 activity by *P. gingivalis* infection in GECs. This finding was consistent with our hypothesis that *P. gingivalis* favors active CD73 signaling to promote intracellular persistence in the host. Additionally, stimulating *P. gingivalis-*infected GECs with AMP, a direct substrate that increases CD73 enzymatic activity, induced higher amounts of intracellular *P. gingivalis* determined by the antibiotic protection assay. This result indicates that more *P. gingivalis* are present within the host cells upon presence of extracellular AMP and the bacteria are replicating at a higher rate compared to the *P. gingivalis* without AMP treatment. The observed increase in quantity of intracellular bacteria was markedly abolished when *P. gingivalis-*infected GECs were further treated with the CD73-specific inhibitor, APCP, signifying CD73 as a key modulator to the shown elevation in bacterial intracellular survival.

We have previously demonstrated that *P. gingivalis* effector enzyme, nucleoside-diphosphate-kinase (ndk), is extracellularly secreted from *P. gingivalis*-infected GECs [8]. This enzyme homolog that converts the nucleotides can hydrolyze eATP, thereby inhibiting eATP/P2X_7_ receptor-mediated ROS production [3,37,40]. Additionally, we have recently published that *P. gingivalis* in GECs not only modulates antibacterial NADPH oxidase-mediated ROS generation but also induces robust host-antioxidant glutathione synthesis to avoid bacterial clearance [39]. These host-adaptive features of *P. gingivalis* in the previous publications suggested that eATP-mediated adenosine signaling might regulate the amount of cellular ROS during the infection. Our results from this study also revealed a novel cellular mechanism where CD73 inhibition results in an increase in ROS production, implicating that active CD73 induced by *P. gingivalis* can be a critical switch of dampening the initial host innate antimicrobial response.

Another key inflammatory mediator that *P. gingivalis* needs to overcome for successful survival is potent pro-inflammatory cytokine production upon microbial infection [55]. We observed that CD73 inhibition promotes robust and sustained cellular ROS accumulation in GECs (Figure 4). Recent studies demonstrated that ROS can activate IL-6 signaling in nerve cells [66] and inhibition of ROS can specifically block IL-6 release in skeletal muscle cells [67]. IL-6 is a pleiotropic pro-inflammatory cytokine that is dysregulated in initial periodontal inflammation and may contribute to dysbiotic host environment [81–84]. As shown in the present study, *P. gingivalis* infection alone can lead to robust pro-inflammatory IL-6 secretion by the GECs in earlier stages of infection. However, active and enhanced CD73 was able to abolish this host-mediated IL-6 production against *P. gingivalis* in GECs, which indicates that the pathogen likely targets anti-inflammatory CD73 signaling to suppress IL-6 levels in the host cell. In addition, our novel results unexpectedly revealed that exogenous treatment of IL-6 can markedly decrease intracellular *P. gingivalis* in GECs, which could be reversed by stimulating CD73 activity by exogenous AMP treatment and additional CD73 overexpression implemented for a greater reversal effect. There have been some studies showing pro-inflammatory cytokines in clinical isolates can alter growth of pathogens such as *Escherichia coli* and *Staphylococcus aureus* [85–87]; however, the studies were performed solely in bacterial growth culture without providing molecular mechanism. The present study for the first time shows that external treatment of a pro-inflammatory cytokine-like IL-6 can alter the intracellular bacterial survival like *P. gingivalis* in human host cells. Additionally, the concentrations of IL-6 that led to the significant decrease in *P. gingivalis* intracellular survival in GECs were similar to the published range during systemic inflammation [88]. Interestingly, a recent report showed that CD73 inhibition (with the same CD73-specific inhibitor, APCP) up-regulates macrophage production of both pro-inflammatory cytokine and nitric oxide, which are required host immune responses for bacterial clearance during salmonellosis [79]. Therefore, it is tempting to speculate that other opportunistic bacteria causing intracellular mucosal infections like *P. gingivalis* may usurp CD73 as described in this study. However, further investigations are needed to fully determine the generality of the current findings.

**Figure 8.**
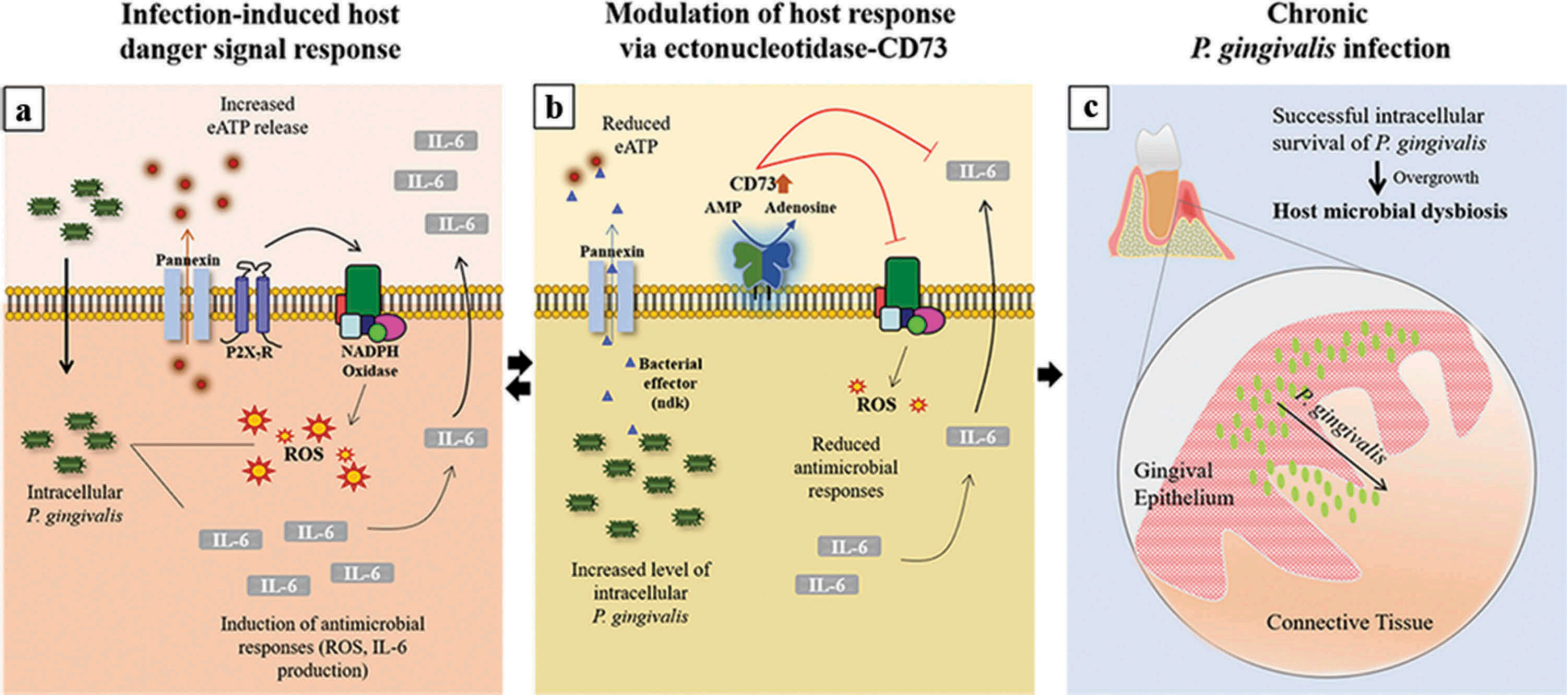
Sequential modulation of the host-danger signaling by the interplay of homeostatic host-enzyme, CD73, and host-adaptive pathogen, *P. gingivalis*, disarms major cellular innate molecules, ROS and IL-6, in human gingival epithelium. a) Upon *P. gingivalis* invasion, extracellular ATP (eATP)-induced antimicrobial Reactive-Oxygen-Species (ROS) formation and Interleukin-6 (IL-6) secretion are induced. b) Extracellular secretion of *P. gingivalis*-nucleoside diphosphate kinase (ndk), acting as an autocrine effector to the host, modulates the P2X_7_-receptor/pannexin hemichannel (3,8); in parallel, homeostatic CD73 expression and activity is modified by *P. gingivalis* to counteract the innate host responses resulting in inhibition of epithelial ROS and IL-6 generations. c) Thus, these dysregulated molecular events create a favorable intracellular environment for *P. gingivalis* in successful evasion of the epithelial antibacterial intracellular immunity, thereby establishing long-term microbial survival. The eventual overgrowth of *P. gingivalis* and its chronic infection in GECs later might lead to host microbial dysbiosis in oral mucosa.

In summary, our novel findings showed that CD73, a host-protective surface molecule that majorly contributes to eATP danger-molecule enzymatic cascade, can be targeted by *P. gingivalis* for the enhanced host intracellular symbiosis resulting in robust CD73 expression and activity in epithelial cells. Moreover, we identified potentially a new homeostatic function of the ectonucleotidase-CD73 in host cells for attenuating potent antimicrobial responses such as ROS inhibition and IL-6 secretion, which ultimately supports opportunistic organisms such as *P. gingivalis* to sustain chronic presence in the epithelial cells (Figure 8). By identifying IL-6 as an important cytokine for *P. gingivalis* infection in the epithelial cells, along with a newly discovered role of CD73 in epithelial IL-6 biology, we propose the described interactions can present a significant advantage for *P. gingivalis’* intracellular persistence in the epithelial cells. Thus, the findings here display a distinct host-microbe adaptation strategy that may contribute to microbial dysbiosis in oral mucosa and might be operational for other chronic bacteria.

## Materials and methods

### Bacterial and eukaryotic cell culture

*P. gingivalis* ATCC strain 33277, which has been shown to be more invasive in the intracellular infection models compared to other relevant strains (i.e. W83 and 381) [89–92], was used for this study. *P. gingivalis* ATCC strain 33277 was anaerobically cultured at 37°C in Trypticase soy broth (TSB) supplemented with yeast extract (1 mg/ml), hemin (5 μg/ml) and menadione (1 μg/ml). Bacteria were harvested by centrifugation at 6000 *g* and 4°C for 10 min and re-suspended in Dulbecco’s phosphate-buffered saline (PBS), pH 7.3. The bacteria were quantified at mid-log phase using a Klett-Summerson photometer and used at a multiplicity of infection (MOI) of 100 for all assays described below.

Primary human GECs were obtained and cultured as described previously [32,37,39,43,93,94]. Briefly, gingival tissue was collected from healthy adult individuals who were selected anonymously and randomly from those presenting at the University of Florida Dental Clinics for tooth crown lengthening or impacted third molar extraction. No patient information was collected. Gingival tissue that would otherwise be discarded was collected after informed consent was obtained by all patients under the approved guidance of the University of Florida Health Science Center Institutional Review Board (IRB, human subjects assurance number FWA 00005790). Cells were cultured in serum‐free keratinocyte growth medium (KGM, Lonza) at 37°C in 5% CO_2_. To assure consistency, only the early passage numbers of the primary GECs were utilized for experimentation, which were repeated using cells derived from multiple patient samples.

### Western blotting analysis for CD73 expression

CD73 protein expression in GECs was evaluated via Western blotting as previously performed [43,63,68]. Cell lysates of GECs infected with *P. gingivalis* for 1, 6, and 24 h were collected in 1x RIPA buffer with protease inhibitor cocktail (Thermo Fisher) and protein levels were determined by Bradford assay. The cell lysates in 1x Laemmli Sample Buffer were loaded onto a 10% SDS-Page gel at 140 V for 1 h. The separated proteins were transferred to a nitrocellulose membrane via wet transfer at 80 V for 1 h and blocked with 5% nonfat dry milk in Tris-buffered saline and 0.1% Tween-20. The membrane was incubated overnight at 4°C in 1:1,000 mouse CD73 (Abcam) and mouse GAPDH (Abcam) antibodies, washed, and incubated with secondary mouse HRP conjugated antibody for 1 h. Protein bands were visualized using enhanced chemiluminescence (GE Healthcare) and analyzed using NIH ImageJ software.

### Confocal microscopy

Cells were infected with *P. gingivalis* 33277 for 1, 6, or 24 h, fixed using 10% neutral buffered formalin, blocked with 3% BSA, and stained for 1 h with a 1:50 mouse CD73 antibody (Abcam) and a custom-made 1:1,000 rabbit *P. gingivalis* 33277 antibody (Pacific Immunology, Ramona, CA). The stained cells were washed and incubated for another 1 h with Alexa Fluor 568 conjugated secondary goat anti-mouse antibody and Alexa Fluor 488 conjugated secondary goat anti-rabbit antibody (1:1,000; Invitrogen). Fixed cells were mounted using VectaShield mounting medium containing DAPI (Vector Laboratories). Images were acquired and processed at 63x objective with oil using Zeiss LSM 880 Quasar NLO multiphoton/confocal system with Zeiss Zen Microscope Software.

### CD73 activity assay

The enzymatic activity of CD73 was determined by the release of inorganic phosphate (Pi) as previously described [95,96]. Briefly, GECs pre-incubated in phosphate-free buffer (2 mM MgCl2, 125 mM NaCl, 1 mM KCl, 10 mM glucose, 10 mM Hepes pH 7.2, diluted in ddH2O) were treated with α,β-methylene adenosine-5′-diphosphate (APCP) (Sigma), a CD73-specific inhibitor, diluted in phosphate-free buffer [50 μM; unless indicated otherwise]. The cells were incubated with AMP [100 μM, unless indicated otherwise] (Sigma) diluted in phosphate-free buffer for 30 min and the enzymatic reaction was terminated on ice for 10 min. Cell culture supernatant was collected to study the Pi release using Malachite Green Phosphate assay kit (BioAssay Systems) according to the manufacturer’s protocol with a BioTek H1 M monochromatic plate reader at 620 nm. Non-enzymatic hydrolysis was measured by substrate solution without cells. The baseline Pi release for each condition was determined by adding blank buffer. For the determination of kinetic parameters, the AMP and APCP concentrations were selected based on previously used range [97] as well as our results showing no cytotoxic effect on GECs with the chosen concentrations.

### IL-6 qRT-PCR

Transcription of *IL-6* was measured by qRT-PCR, using the SybrGreen detection system using the following pairs of primers: human-specific *IL-6* (Forward: 5ʹ- GTAGCCGCCCCACACAGA-3ʹ; Reverse: 5ʹ-CATGTCTCCTTTCTCAGGGCTG-3ʹ) and human-specific GAPDPH (Forward: 5ʹ-GAAATCCCATCACCATCTTCCAGG-3ʹ; Reverse: 5′-GAGCCCCAGCCTTCTCCATG-3′) [98]. Total RNA was extracted with Trizol (Invitrogen) and 1 μg of the total RNA per sample was reverse-transcribed using a High Capacity cDNA Reverse Transcription Kit (Applied Biosystems). qPCR was conducted at 95°C for 3 min, followed by 45 cycles at 95°C for 30 s and either 55°C (for *IL-6* and GAPDH) for 30 s. Gene expression analysis was performed using the CFX Manager Software (BioRad). The measured expression of GADPH was used as a reference gene. For each condition, uninfected GECs were assigned as control.

### IL-6 ELISA

GECs were incubated with AMP [100 μM] and/or APCP [50 μM] 1 h and 30 min, respectively, and infected with *P. gingivalis*. For CD73 depleted and overexpressing GECs, the transfection described below was performed first for at least 24 h. Cell culture supernatants were collected after selected timepoints of *P. gingivalis* infection. Uninfected and/or untreated GECs were used as controls. The quantitative measurement of human IL-6 by ELISA was performed following the manufacturer’s protocol (R&D systems). The optical density was read at absorbance of 450 nm using a Biotek H1 M monochromatic plate reader with wavelength correction of 540 nm. A standard curve was obtained from 600 to 9.38 pg/mL. No impact on the initial bacterial invasion by AMP and APCP treatment was confirmed prior to this assay.

### Antibiotic protection assay

*P. gingivalis* intracellular survival in GECs was determined as described previously [63]. Briefly, GECs were incubated with AMP [100 μM] and/or APCP [50 μM] 1 h and 30 min, respectively, and infected with *P. gingivalis* for 6 or 24 h. GECs were washed with PBS and treated with gentamicin (300 µg/mL) and metronidazole (200 µg/mL) for 1 h. Total RNA was isolated using Trizol Reagent (Invitrogen). Genomic DNA contamination was removed by DNase digestion (Ambion). cDNA was synthesized from 1 μg Total RNA using High Capacity cDNA Reverse Transcriptase Kit (Applied Biosystems). A 1:10 cDNA was used to detect *P. gingivalis* 16s rRNA by SYBR Green Real-time qPCR (Forward: 5ʹ-TGTAGATGACTGATGGTGAAAACC-3ʹ; Reverse: 5ʹ-ACGTCATCCCCACCTTCCTC-3ʹ) [99]. qPCR was performed in CFX96 real-time system (Bio-Rad) with an initial cycle of 98°C for 3 min followed by 40 cycles of 9 5°C for 15 s, 60.7°C for 30 s, and 72°C for 30 s. The CFU of *P. gingivalis* in unknown sample was calculated from the standard curve prepared according to the published method. In brief, *P. gingivalis* was grown to early log phase (OD_600_ = 0.2) and 1 ml of culture was used for CFU count by 10-fold serial dilution followed by plating on TSB supplemented with yeast extract (5 mg/ml), hemin (5 μg/ml) and menadione (1 μg/ml), agar (1.5%) and sheep blood (5%). Another 1 ml was used to isolate genomic DNA and 10-fold serial dilution of DNA was used for qPCR using the primers and conditions mentioned here. A standard curve was prepared with the C_t_ (Threshold cycle) value obtained from qPCR and CFU counted on agar plates (correlation coefficient = 0.999). This standard curve was then used to calculate CFU of corresponding Ct value obtained from qPCR performed with cDNA of each infected-GEC condition (as we published previously [63]).

### *Silencing of CD73 (*NT5E*) by small interfering RNA*

GECs were transfected with ON-TARGETplus Human *NT5E* siRNA (Dharmcon) or Control siRNA (Life Technologies) using Lipofectamine™ RNAiMax Protocol (Invitrogen) for 24 or 48 h. Cell lysates were collected and the silencing was confirmed by Western blotting as described previously in this study.

### *Transient transfection with pcDNA‑*NT5E *plasmid for CD73 overexpression*

pcDNA3.0+ expression vector with CD73 coding gene (*NT5E*) insert (GenScript) was transfected into human primary GECs using Lipofectamine™ 3000 reagent (Invitrogen) following the manufacturer’s protocol. pcDNA3.0+ vector without *NT5E* insert (empty vector) was used as a control. Confirmation of CD73 overexpression in GECs was done by Western blotting as described previously in this study using Flag antibody (1,1:000; Cell Signaling).

### Host cell ROS production measurement

Cellular ROS production in GECs was determined as described previously [3,39]. Briefly, GECs were pre-stimulated with ATP [3 mM] and further incubated with 50 μM APCP. GECs were labeled with the fluorescent probe 5-(and-6)-chloromethyl-2′,7′-dichlorodihydrofluorescein diacetate, acetyl ester (CM-H_2_DCFDA) (Invitrogen) which was solubilized in dimethyl sulfoxide (Sigma) and diluted to 5 μM in Hanks balanced salt solution (HBSS) containing MgCl_2_ and CaCl_2_ (Invitrogen). The CM-H_2_DCFDA (green) fluorescence intensity was measured using a Biotek H1 M monochromatic bottom fluorescence plate reader at excitation 495 nm and emission 525 nm. The representative live images of CM-H2DCFDA fluorescence intensity were taken using epifluorescence (DM IRE2 HC inverted scope, Leica Microsystems GmbH) microscopy equipped with DIC. The single exposure images were collected sequentially in fluorescence and DIC using a Retiga 4000 r CCD camera (Qimaging). Diphenyleneiodonium (DPI), a broad-spectrum NADPH oxidase inhibitor was used as a positive control.

### Statistical analysis

All experiments were performed in at least three separate occasions with technical duplicates or triplicates. The results were initially analyzed by one-way ANOVA and then by two-tailed Student’s *t*-test. *P-*values determined by the *t-*test are reported while values of 0.05 or less were considered statistically significant.
