# Supplementary material for: Host surface ectonucleotidase-CD73 and the opportunistic pathogen, Porphyromonas gingivalis, cross-modulation underlies a new homeostatic mechanism for chronic bacterial survival in human epithelial cells
Source: Virulence. 2020 May 18;11(1):414–29. doi: 10.1080/21505594.2020.1763061 (PMC7239027; doi:10.1080/21505594.2020.1763061)
Supplement: Supplemental Material [file kvir-11-01-1763061-s001.zip › suppl caption.docx]

**Figure S1. Additional images showing an increase in CD73 expression in GECs during *P. gingivalis i*nfection.** Further representative images are shown to support the data in Figure 1C and 1D. **A)** Cells with *P. gingivalis* infection over 24h were fixed, stained with primary antibody against CD73 and then secondary antibody (Alexa fluor 468; red), and mounted with DAPI (blue) to visualize nuclei. Representative images were obtained via confocal microscopy at 63x objective with oil immersion. **B)** Confocal micrographs showing orthogonal views of surface CD73 expression in GECs.

**Figure S2. IL-6 mRNA levels increase at 6 h of *P. gingivalis* infection in GECs.** Cells were infected with *P. gingivalis* (MOI 100) for 1, 6, and 24h. Total mRNA was isolated, and PCR was performed as described in the Materials and Methods. **A)** The PCR products were then analyzed by agarose gel electrophoresis and ethidium bromide staining using EconoTaq PLUS GREEN 2X Master Mix (Lucigen, Middleton, WI) on a 2% agarose gel at 100v for 30min. **B)** Quantification of the PCR amplicons of *IL-6* presented in A) normalized to GADPH as a housekeeping gene.

**Figure S3. *P. gingivalis* infection to host GECs has no impact on cell viability up to 24 h.** Cells were incubated with *P. gingivalis* (ATCC strain 33277; MOI 100) over 24 h. GEC cell viability was assessed using MTT ((3-(4,5-dimethylthiazol-2-yl)-2,5-diphenyltetrazolium bromide) assay. n.s = no statistically significant difference (p > 0.05), Mean ± SEM. N=3.
