# Supplementary figures and images for: Host surface ectonucleotidase-CD73 and the opportunistic pathogen, Porphyromonas gingivalis, cross-modulation underlies a new homeostatic mechanism for chronic bacterial survival in human epithelial cells
Source: Virulence. 2020 May 18;11(1):414–29. doi: 10.1080/21505594.2020.1763061 (PMC7239027; doi:10.1080/21505594.2020.1763061)

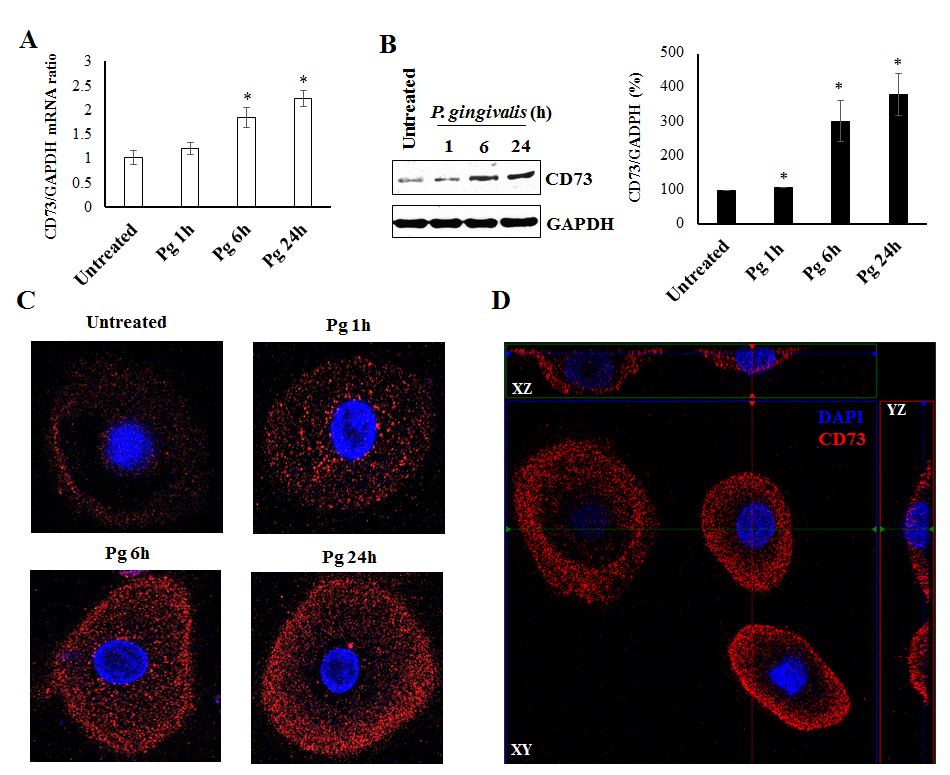

Supplement: Supplemental Material [file kvir-11-01-1763061-s001.zip › Supplemental Figure1 - 500 mpi.tif]

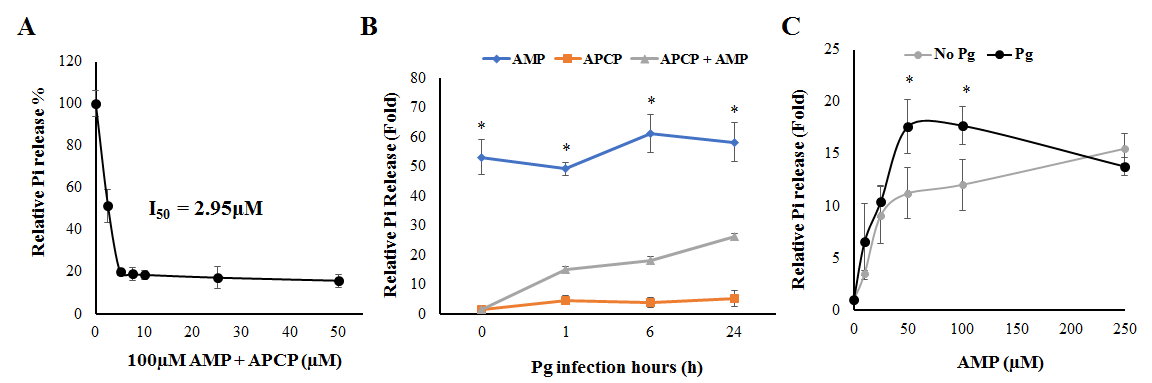

Supplement: Supplemental Material [file kvir-11-01-1763061-s001.zip › Supplemental Figure2 - 500 mpi.tif]

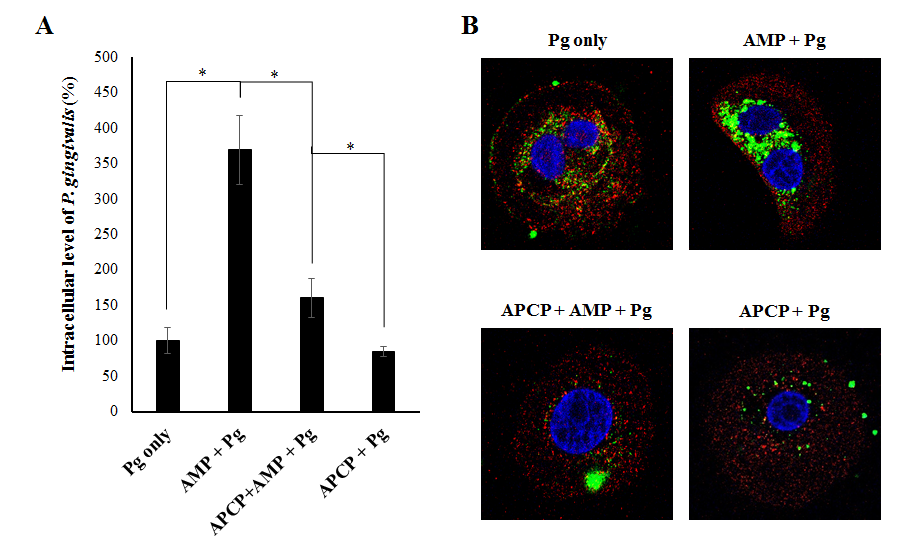

Supplement: Supplemental Material [file kvir-11-01-1763061-s001.zip › Supplemental Figure3 - 500 mpi.tif]
